# Supplementary material for: MAIT cells protect against pulmonary Legionella longbeachae infection
Source: Nat Commun. 2018 Aug 22;9:3350. doi: 10.1038/s41467-018-05202-8 (PMC6105587; doi:10.1038/s41467-018-05202-8)
Supplement: Supplementary file 1 — Supplementary Information [file 41467_2018_5202_MOESM1_ESM.pdf]

Remove Watermark Now

Supplementary information for:

Mucosal Associated Invariant T (MAIT) cells protect against pulmonary *Legionella longbeachae* infection

by **Wang H *et al.***

pdfelement

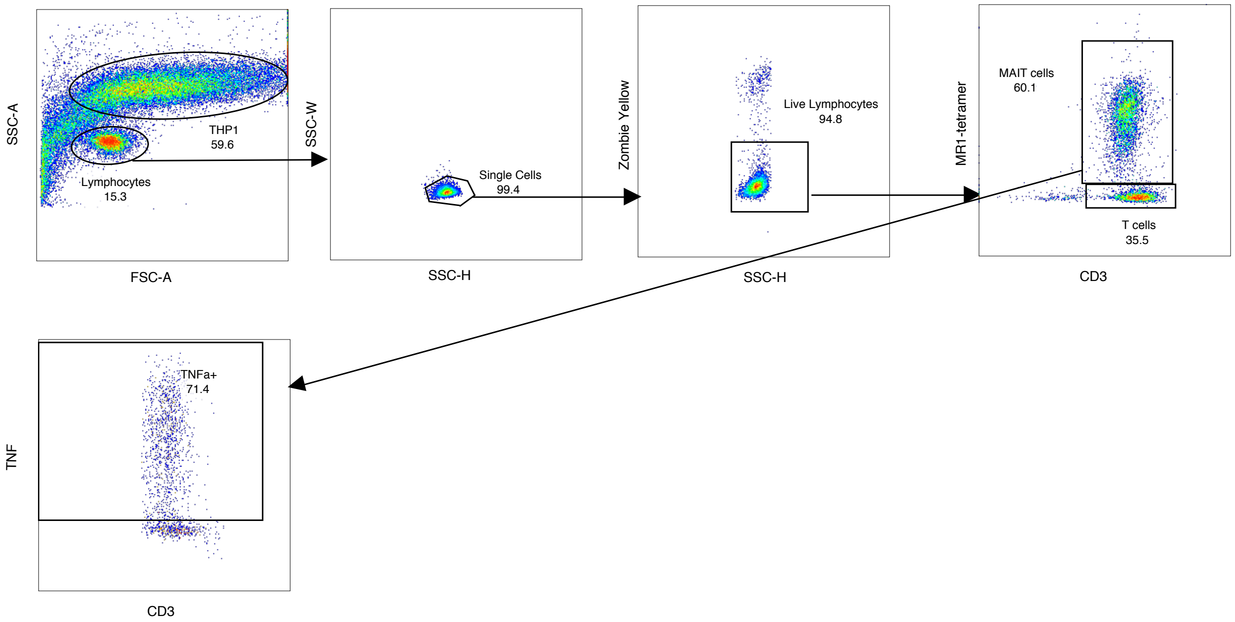

Supplementary figure 1. Related to Figure 1B, C. Cytometric gating strategy for human MAIT cells *in vitro*.

pdfelement

Flow-sorted human peripheral blood $CD3^{+}V\alpha 7.2^{+}CD161^{+}$ MAIT cells or conventional T cells

were co-cultured for 16 h with THP1 cells infected 27 h previously with live or heat-killed *L. longbeachae.* After co-culture MAIT cells were identified by gating on the lymphocyte population and excluding doublets using forward scatter / side scatter properties; Zombie Yellow-negative live lymphocytes are gated and MAIT cells further selected as co-staining for CD3 and MR1-5-OP-RU tetramer and cytokine secretion measured by intracellular cytokine staining.

Remove Watermark Now

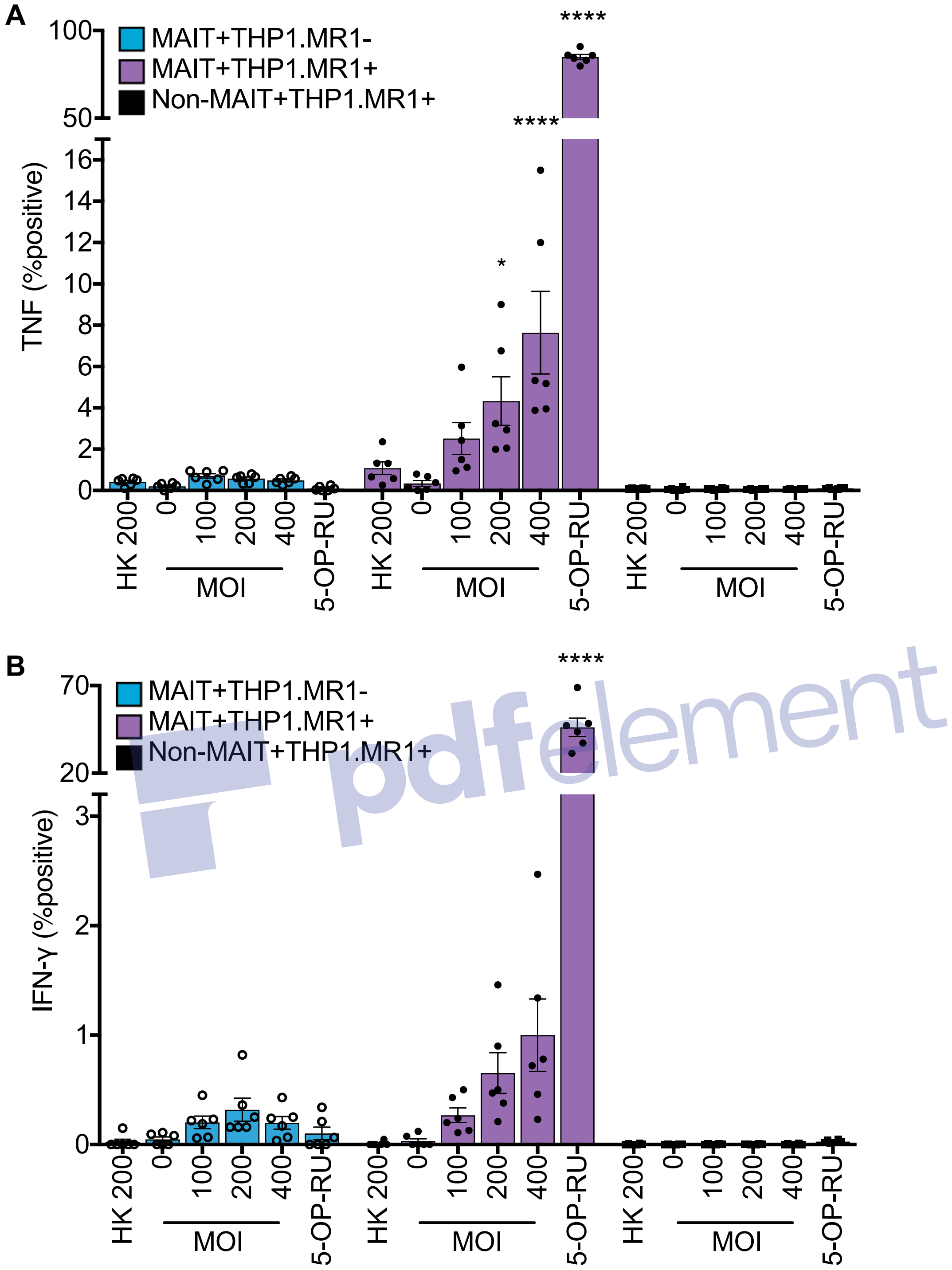

pdfelement

Supplementary figure 2. Related to Figure 1b, c. Human MAIT cells are activated by *Legionella* infection via MR1 *in vitro*.

Remove Watermark Now

THP1 cells overexpressing MR1 ($THP1.MR1^+$, purple) or deficient in expression of MR1 ($THP1.MR1^-$, blue) were infected for 3h with *L. longbeachae* (MOI 100, 200, 400), heat killed (HK) *L. longbeachae* (MOI 200) or left untreated (Nil), or with 10nM 5-OP-RU, then treated with gentamicin to kill extracellular bacteria, and co-cultured with human PBMCs for 6h, in the presence of Brefeldin A. MR1-5-OP-RU-tetramer$^-$ non-MAIT cell activation was measured by intracellular cytokine staining for (**A**) TNF or (**B**) IFN-γ. Data is from a separate experiment from figure 1B, C with mean + SEM from 6 donors, where each dot represents 1 donor. Statistics: One-way ANOVA with Sidak's multiple comparisons test, ****$p<0.0001$.

pdfelement

Remove Watermark Now

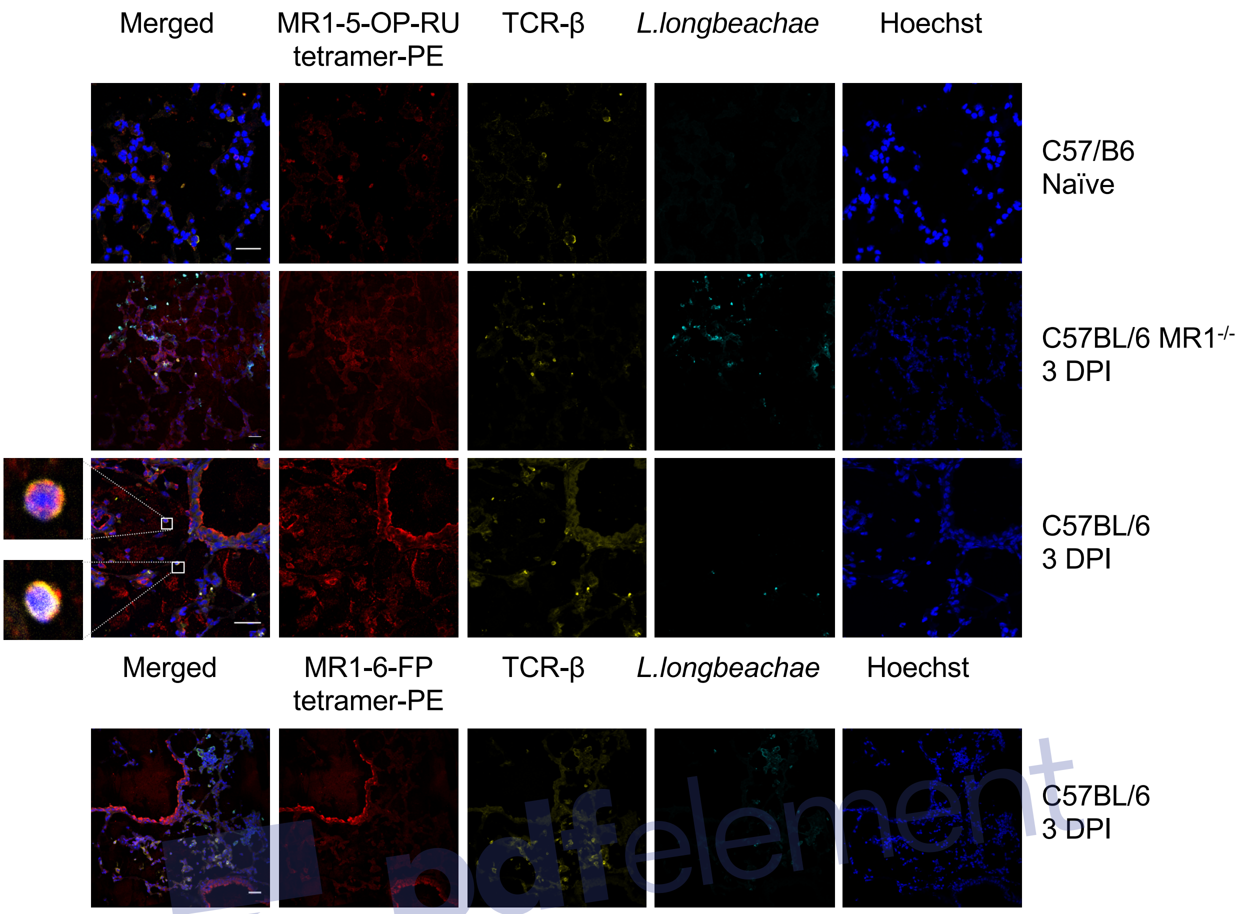

pdfelement

Supplementary figure 3. Related to Figure 2b. Murine MAIT cells visualised by MR1-5-OP-RU tetramer in the lung parenchyma during *L. longbeachae* infection.

Immunofluorescence micrographs of murine lungs showing TCRβ$^+$, MR1-5-OP-RU-tetramer$^+$ MAIT cells (white boxes) in *L. longbeachae*-infected C57BL/6 mice (panel 3, 3 days post infection (DPI)). Images from Figure 2b are shown in the context of their relevant controls. Control mice were naïve C57BL/6 (panel 1) and MR1$^{-/-}$ mice (panel 2, 3 DPI). Lung cells from 3-day infected C57BL/6 mice were also stained with control tetramer MR1-6-FP (panel 2, row 4). All infections were delivered intranasally with $2x10^4$ CFU *L. longbeachae.* Red, MR1-5-OP-RU tetramer (panels 1-4) and MR 1-6-FP negative control tetramer (panel 5); yellow, TCR-β; cyan , *L. longbeachae* ; blue, nuclei (Hoechst). Scale bars are 30 μm.

Remove Watermark Now

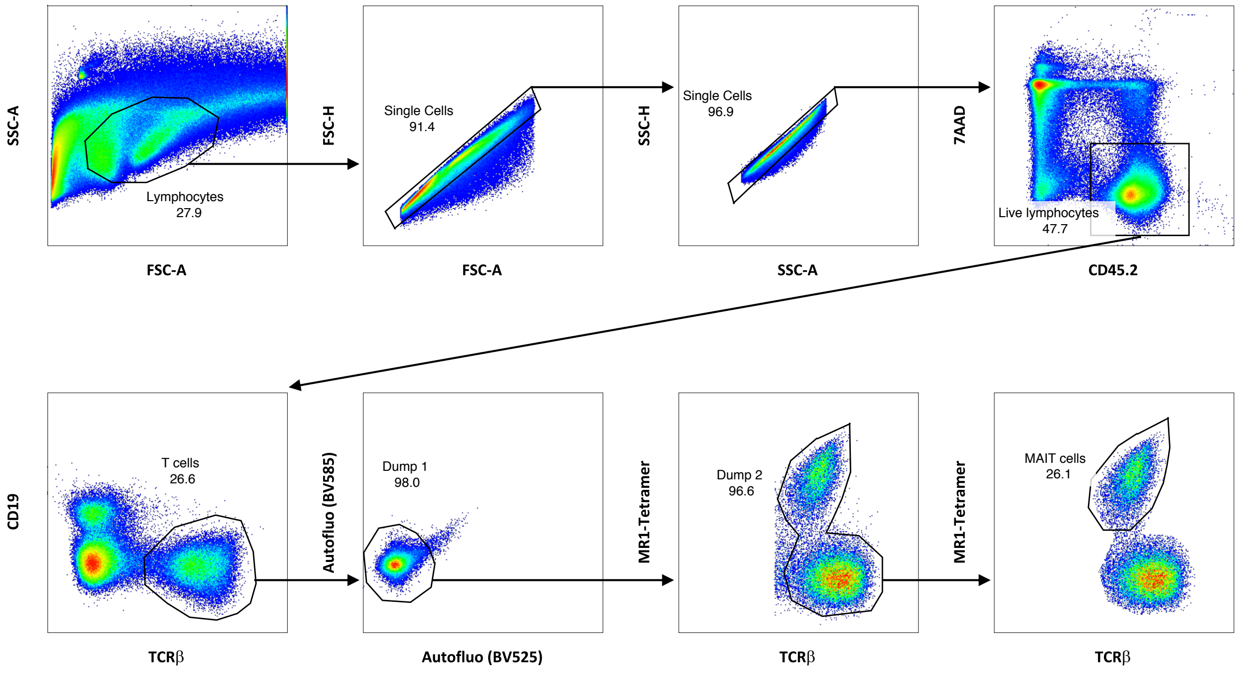

Supplementary figure 4 Related to Figure 3. Cytometric gating strategy for murine MAIT cells.

Murine pulmonary MAIT cells are identified by gating on the lymphocyte population and excluding doublets using forward scatter / side scatter properties; 7AAD$^-$ CD45$^+$ live lymphocytes are gated and further selected as CD19$^-$ TCR-β$^+$; autofluorescent cells are excluded using fluorescence in BV525 and BV585 channels and TCR-β$^+$ T cells or TCR-β$^+$MR1-5-OP-RU tetramer$^+$ MAIT cells are then gated as shown.

pdfelement

Remove Watermark Now

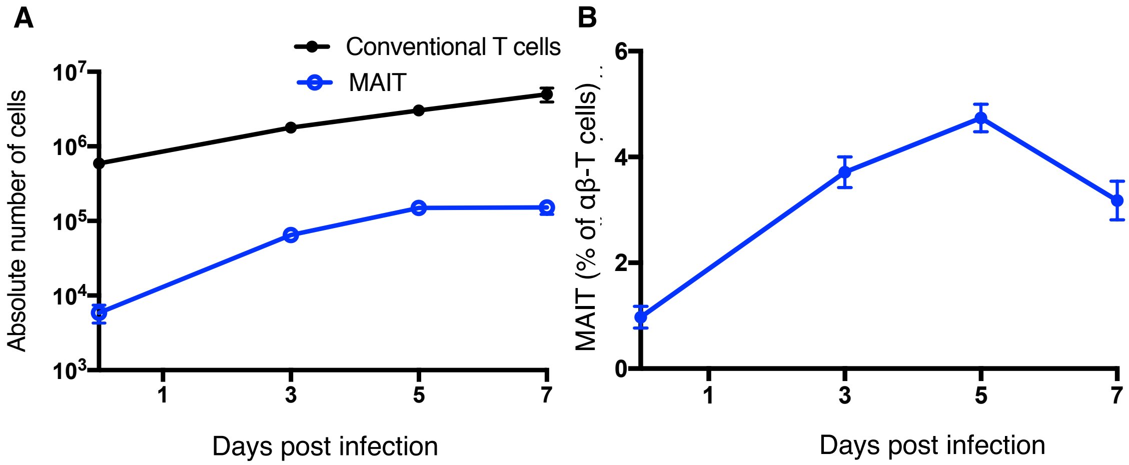

Supplementary figure 5. Related to Figure 3. MAIT cells expand after pulmonary infection with *Legionella pneumophila* in mice

(**A**) Absolute numbers of MR1-tetramer+ MAIT cells (blue line) or conventional αβ T cells (black line) in C57BL/6 mice at various time points after intranasal infection with $2x10^7$ CFU *L. pneumophila.* (**B**) Relative frequencies of MAIT cells as a percentage of αβ T cells in the same mice. Pooled data were shown from two experiments with seven to nine mice per time point (mean±SEM).

Remove Watermark Now

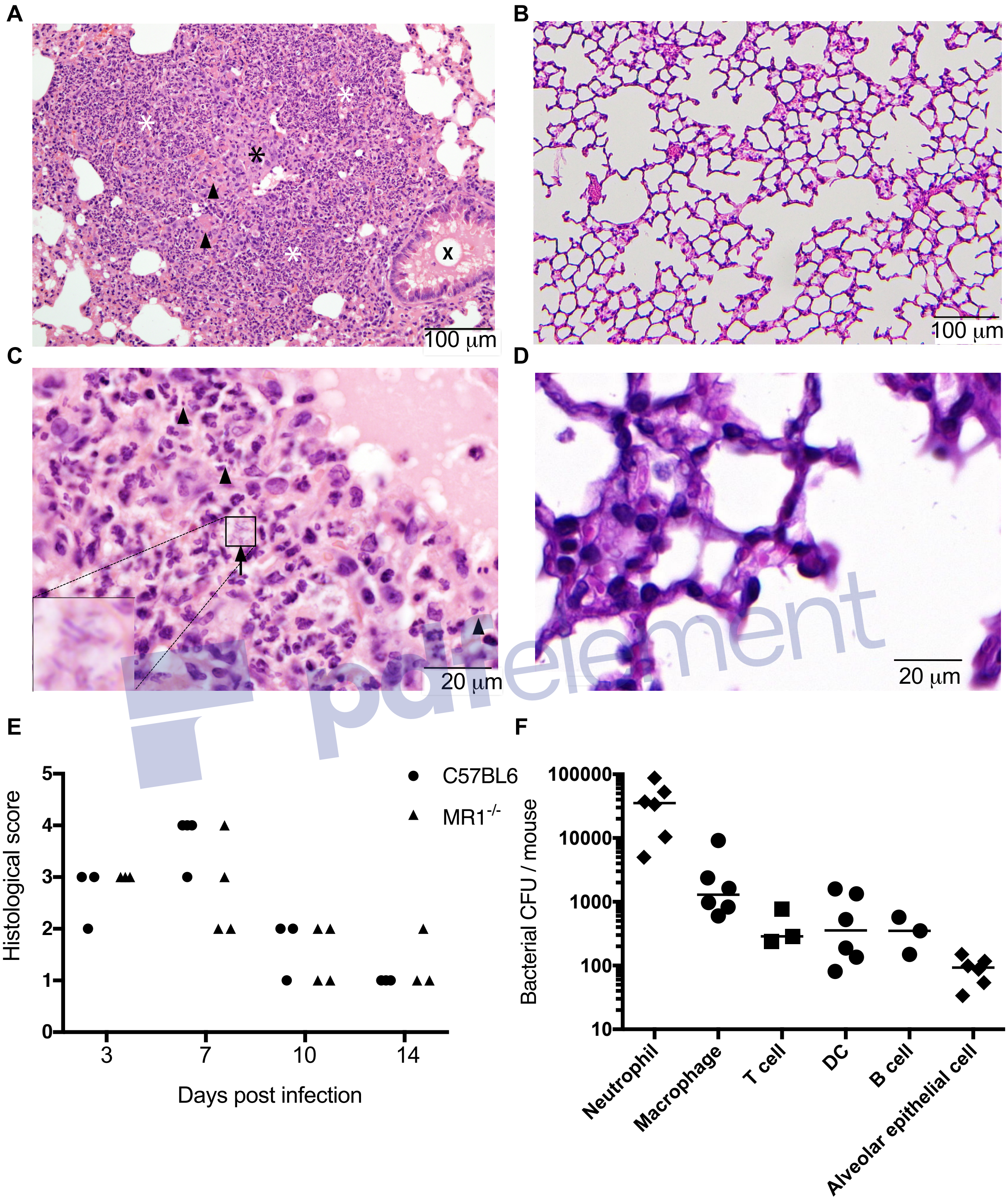

pdfelement

Remove Watermark Now

Supplementary figure 6. Related to Figure 3. Histological changes associated with pulmonary *Legionella* infection in mice

(**A,C**) Photomicrographs of haematoxylin and eosin-stained sections of lung lesions from C57BL/6 mice infected i.n. with $2x10^4$ CFU *L. longbeachae* at day 7 post infection. (**A**) Displays alveoli filled with neutrophils (white asterisks) and a focal cluster of macrophages (black asterisk). Aggregates of fibrin are also present (arrowheads). There is accumulation of oedema fluid and mild epithelial shedding in an adjacent bronchiole (black X). (**C**) Detail of region of neutrophilic inflammation displaying nuclear fragmentation (leukocytoclasia, arrowheads) and intracytoplasmic bacillary bacteria consistent with *Legionella* spp. (arrow). (**B**,**D**) uninfected C57BL/6 controls showing normal bronchioles and alveoli. (**E**) Histological grading of pulmonary inflammation at indicated time-points after infection with $2x10^4$ CFU *L. longbeachae* i.n. in C57BL/6 and $MR1^{-/-}$ mice. Sections were graded for severity of inflammation on a scale of 0 to 5 in a double blinded manner by a veterinary pathologist. No significant differences were observed between groups at individual time-points. (**F**) Determination of cellular localization of viable *L. longbeachae* in the murine lung. Collagenase-dispersed lung cells from C57BL/6 mice 3 days after infection with $2x10^4$ CFU i.n. were flow-sorted and serial dilutions plated onto charcoal agar. Infection is expressed as total CFU per mouse for each individual cell type: live neutrophils (CD45+Ly6G+), macrophages ($CD45^+CD64^+F4/80^+$), dendritic cells ($CD45^+CD64^-CD11c^+$), B cells ($CD45^+CD19^+CD11c^-$) and alveolar epithelial cells ($EpCAM^+PCAM^-CD146^-$). Pooled data are from two experiments which gave similar results, each with 3 mice per group.

pdfelement

Remove Watermark Now

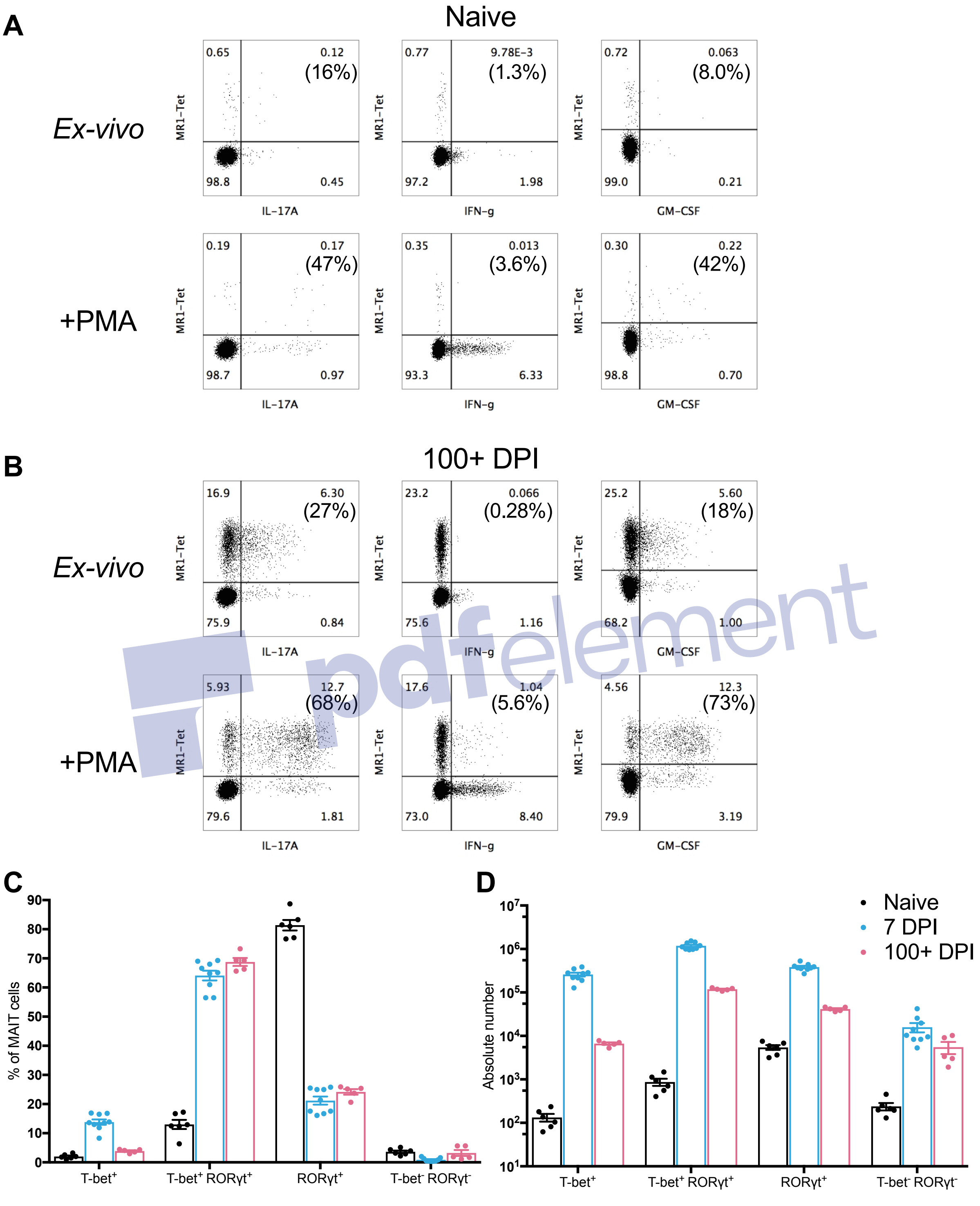

pdfelement

Supplementary figure 7. Related to Figure 4. Cytokine expression profiles and numbers of differentiated subsets of MAIT cells, in naïve mice and after *Legionella* infection.

(**A**) and (**B**); Representative flow cytometry plots showing intracellular staining for IL-17A, IFN-γ and GM-CSF by pulmonary TCRβ lymphocytes (non-MAIT conventional and MAIT cells) after 4h culture with or without PMA and ionomycin in the presence of brefeldin A. TCRβ+ lymphocytes were harvested from lungs of C57BL/6 mice before (naïve, top two panels) or >100 days after infection (100 DPI, two panels of B) with $2x10^4$ CFU *L. longbeachae*. Percentages in brackets represent the proportion of MR1-tetramer positive MAIT cells expressing each cytokine. (**C**) and (**D**); C57BL/6 mice infected for 0, 7 or >100 days with $2x10^4$ CFU *L. longbeachae*. Experiments using 4-7 mice per group (mean±SEM) were performed twice with similar results. Percentage (C) and absolute numbers (D) of T-bet$^+$, double positive (DP), RORγt$^+$ and double negative (DN) MAIT cells from uninfected or infected C57BL/6 mice at indicated dates, are shown. Mean values are representative of 5-8 mice in each group. See also figure 4D, E.

pdfelement

Remove Watermark Now

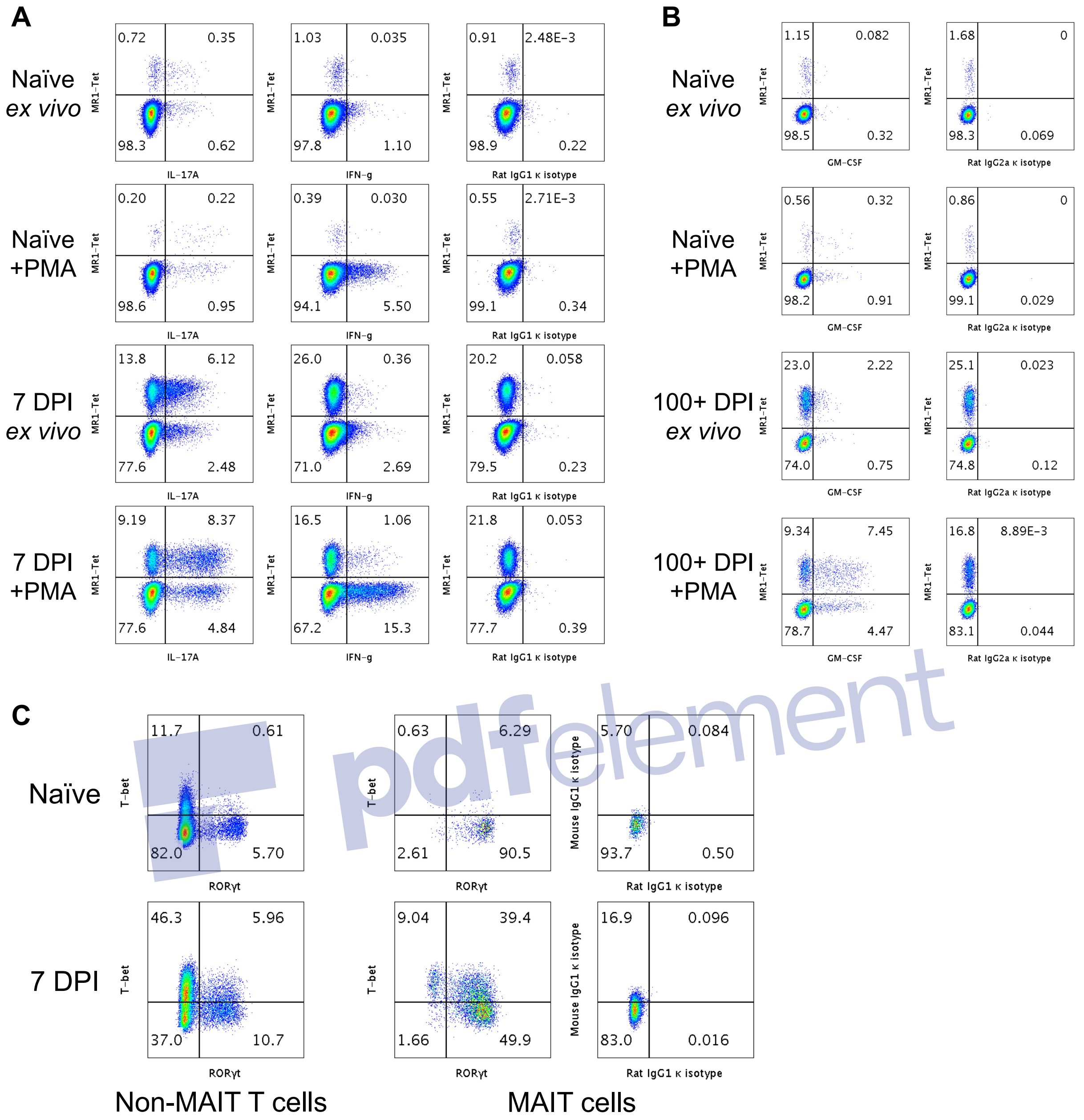

pdfelement

Supplementary figure 8. Related to figure 4, 7 and supplementary figure 7. Basal level staining of isotype controls (right column) for ICS settings for individual cytokine or transcriptional factor profiling.

(**A**) Rat IgG1 isotype control for cytokine profiling of IL-17A and IFN-γ with setting of naïve MAIT cells with/without PMA stimulation or fully activated MAIT cells with/without PMA

stimulation. (**B**) Rat IgG2a isotype control for cytokine profiling of GM-CSF with setting of naïve MAIT cell gates with/without PMA stimulation or fully activated MAIT cells with/without PMA stimulation. (**C**) Rat IgG1 isotype control for transcriptional factor profiling of RORγt and T-bet with setting of naïve and activated MAIT cell gates. Cytokine profiles were validated with isotype controls, alongside with a separate sample stained with isotype controls conjugated with the same fluorescent protein (right column panels). All the antibodies used for cytokine profiling were purchased from BD Pharmingen or eBioscience.

Remove Watermark Now
pdfelement
